# Postconditioning with Inhaled Carbon Monoxide Counteracts Apoptosis and Neuroinflammation in the Ischemic Rat Retina

**DOI:** 10.1371/journal.pone.0046479

**Published:** 2012-09-28

**Authors:** Nils Schallner, Matthias Fuchs, Christian I. Schwer, Torsten Loop, Hartmut Buerkle, Wolf Alexander Lagrèze, Christian van Oterendorp, Julia Biermann, Ulrich Goebel

**Affiliations:** 1 Department of Anesthesiology and Critical Care Medicine, University Medical Center Freiburg im Breisgau, Germany; 2 University Eye Hospital, University Medical Center, Freiburg, Germany; Dalhousie University, Canada

## Abstract

**Purpose:**

Ischemia and reperfusion injury (I/R) of neuronal structures and organs is associated with increased morbidity and mortality due to neuronal cell death. We hypothesized that inhalation of carbon monoxide (CO) after I/R injury (‘postconditioning’) would protect retinal ganglion cells (RGC).

**Methods:**

Retinal I/R injury was performed in Sprague-Dawley rats (n = 8) by increasing ocular pressure (120 mmHg, 1 h). Rats inhaled room air or CO (250 ppm) for 1 h immediately following ischemia or with 1.5 and 3 h latency. Retinal tissue was harvested to analyze Bcl-2, Bax, Caspase-3, HO-1 expression and phosphorylation of the nuclear transcription factor (NF)-κB, p38 and ERK-1/2 MAPK. NF-κB activation was determined and inhibition of ERK-1/2 was performed using PD98059 (2 mg/kg). Densities of fluorogold prelabeled RGC were analyzed 7 days after injury. Microglia, macrophage and Müller cell activation and proliferation were evaluated by Iba-1, GFAP and Ki-67 staining.

**Results:**

Inhalation of CO after I/R inhibited Bax and Caspase-3 expression (Bax: 1.9±0.3 vs. 1.4±0.2, p = 0.028; caspase-3: 2.0±0.2 vs. 1.5±0.1, p = 0.007; mean±S.D., fold induction at 12 h), while expression of Bcl-2 was induced (1.2±0.2 vs. 1.6±0.2, p = 0.001; mean±S.D., fold induction at 12 h). CO postconditioning suppressed retinal p38 phosphorylation (p = 0.023 at 24 h) and induced the phosphorylation of ERK-1/2 (p<0.001 at 24 h). CO postconditioning inhibited the expression of HO-1. The activation of NF-κB, microglia and Müller cells was potently inhibited by CO as well as immigration of proliferative microglia and macrophages into the retina. CO protected I/R-injured RGC with a therapeutic window at least up to 3 h (n = 8; RGC/mm^2^; mean±S.D.: 1255±327 I/R only vs. 1956±157 immediate CO treatment, vs. 1830±109 1.5 h time lag and vs. 1626±122 3 h time lag; p<0.001). Inhibition of ERK-1/2 did not counteract the CO effects (RGC/mm^2^: 1956±157 vs. 1931±124, mean±S.D., p = 0.799).

**Conclusion:**

Inhaled CO, administered after retinal ischemic injury, protects RGC through its strong anti-apoptotic and anti-inflammatory effects.

## Introduction

Stroke, an ischemic cerebral injury, is a leading cause of morbidity and mortality in the Western world and may occur in the perioperative period [1]. Perioperative stroke is primarily associated with major cardiovascular procedures but has also been reported after non-cardiac surgery, occurring with an incidence of 0.1% [2]. Pre-clinical evaluation of many neuroprotective strategies showed only modest or inconsistent tissue protection [3].

The gas carbon monoxide (CO), which is generated in cells almost exclusively through the degradation of heme by heme oxygenase (HO) enzymes, has been shown to protect cells through potential anti-inflammatory, anti-proliferative, or anti-apoptotic effects [4]–[6]. Moreover, CO *preconditioning* has been shown to protect neuronal cells in the brain [7] and the retina [8]. These protective effects of CO involve the modulation of numerous cellular targets including heme-containing enzymes [9], the mitogen-activated protein kinases (MAPKs) [10] and different transcription factors [11], [12]. The MAPKs (p38, ERK-1/2 and JNK) are a family of protein kinases playing an important role in apoptosis and survival signaling. Depending on stimulus and timing, their activity is differentially regulated by CO [4], [8].

Inhalation of 125 or 250 ppm CO immediately at the onset of reperfusion reduced total hemispheric infarct volume in transient middle cerebral artery occlusion model by nearly 30% and 60%, respectively, with an extended therapeutic window of 1–3 h after ischemia [13]. However, the effects and the mechanisms of CO postconditioning on neuronal cells *in vivo*, in particular on retinal ganglion cells (RGC), have not been investigated. The RGC represent a special population of neuronal cells, as they are positioned “upstream” of the central nervous system, easily accessible and treatable under visual control. They are often used as an ischemia/reperfusion (I/R) brain injury model to prove neuroprotective strategies [14]–[16]. Therefore, we chose the eye as a neuronal organ to analyze and counteract I/R related neuronal damage.

The hypothesis of this study was that CO *postconditioning* exerts protective effects over a time period of seven days after retinal ischemia. Furthermore, we hypothesized that CO acts as a neuroprotective messenger *in vivo* via its anti-apoptotic and anti-inflammatory effects.

## Materials and Methods

### Animals and Ethics Statement

Adult male and female Sprague-Dawley rats (1∶1, 280–350 g bodyweight, Charles River, Sulzfeld, Germany) were used. Animals were fed with standard rodent diet *ad libitum* while kept on a 12-h light/12-h dark cycle. All procedures involving the animals concurred with the statement of ‘The Association for Research in Vision and Ophthalmology’ for the use of animals in research and were approved by the ‘Committee of Animal Care of the University of Freiburg’ (Permit Number: 35-9185.81/G-11/81). All procedures were performed under adequate anesthesia/analgesia and all efforts were made to minimize suffering. The number of animals used for RGC quantification and molecular analysis was n = 8 per group and time point. For analysis of mRNA and protein expression retinal tissue was harvested at t = 12, 24, 48 and 72 h after CO inhalation.

### Retrograde labeling of RGC

Rats were anesthetized with isoflurane and placed in a stereotactic apparatus (Stoelting, Kiel, Germany) and Fluorogold (FG, 7.8 µl; Fluorochrome, Denver, CO) dissolved in 10% dimethylsulfoxide in PBS was injected into both superior colliculi as described previously [17]. To ensure proper RGC labeling, animals were allowed seven days for retrograde transport of FG before further experimental intervention.

### Retinal ischemia/reperfusion injury and carbon monoxide treatment

Retinal ischemia/reperfusion injury of the rats was performed for 1 hour as previously described after intraperitoneal anesthesia with xylazine and ketamine [8]. Rats without immediate recovery of retinal perfusion at the end of the ischemic period or those with lens injuries were excluded from the investigation, since the latter prevents RGC death and promotes axonal regeneration [18]. To evaluate a neuroprotective effect of inhaled CO, animals were randomized to receive treatment either with room air or with room air supplemented with 250 ppm CO (Air Liquide, Kornwestheim, Germany) for 1 hour in an air-sealed chamber immediately following retinal I/R injury, 1,5 h or 3 h after initiation of reperfusion. A fifth group received ERK-1/2-inhibitor PD98059 (2 mg/kg BW via the tailvene, dissolved in DMSO) before initiation of retinal ischemia and subsequent CO inhalation, a sixth group received PD98059 before retinal ischemia without CO postconditioning.

### RGC quantification

Animals were sacrificed 7 days after ischemia. After whole-mount preparation, densities of FG-positive RGC were determined with a fluorescence microscope (AxioImager; Carl Zeiss, Jena, Germany) and the appropriate bandpass emission filter (FG: excitation/emission, 331/418 nm), as previously described [19]. Briefly, we photographed 3 standard rectangular areas (measuring 0.200 mm×0.200 mm = 0.04 mm^2^ each) at 1, 2 and 3 mm from the optic disc in the central region of each retinal quadrant. Thus, we counted an area of 12×0.04 mm^2^ = 0.48 mm^2^ per retina. Assuming an average retinal area of about 50 mm^2^ in rats [20], we evaluated about 1% of the retina. To determine the number of cells per square millimeter, we multiplied the number of analysed cells/0.04 mm^2^ by 25. Secondary FG-stained activated microglia cells after RGC phagocytosis were separated by morphologic criteria and were excluded from quantification. All averaged data in the text are presented as mean RGC density (cells/mm^2^) ± standard deviation (SD).

### Immunohistochemical staining (DAPI, GFAP, ERK-1/2 and Thy-1, Ki-67, Iba-1)

Immunohistochemistry was performed to evaluate the expression pattern of glial, neuronal, inflammatory and survival promoting proteins in the retina 48 h after CO inhalation (*‘postconditioning’*). Rat eyes (n = 2 per group) were enucleated and immediately fixed in 4% paraformaldehyde overnight at 4°C. Immunohistochemistry was performed according to standardized protocols with monoclonal antibodies against glial fibrillary acidic protein (GFAP; dilution 1∶400; Sigma, Taufkirchen, Germany), ionized calcium binding adaptor molecule 1 (Iba-1; dilution 1∶150, Wako, Neuss, Germany), Thy-1.1 (CD90; dilution 1∶50; Serotec, Duesseldorf, Germany), pERK-1/2 (#4370; dilution 1∶800; Cell Signaling Technology, Danvers, MA, USA) and Ki-67 antigen (dilution 1∶100, BD Biosciences, Heidelberg, Germany), which were then conjugated with their corresponding secondary antibody (Cy2™; green fluorescence; dilution 1∶200; Jackson ImmunoResearch, West Grove, PA, USA or rhodamin; red fluorescence; dilution 1∶50; KPL, Gaithersburg, MD, USA). The nuclei of cells in the retina were stained with 4′,6-diamino-2-phenylindole dihydrochloride hydrate (DAPI, Sigma, Taufkirchen, Germany) added to the embedding medium (Mowiol; Calbiochem, San Diego, CA, USA). Slides were examined under a fluorescence microscope (Axiophot; Carl Zeiss, Jena, Germany).

### Western blot analysis

Retinal tissue for analysis of protein expression was harvested at four different time points (t = 12, 24, 48 and 72 h). Total protein from ¾ of each retina was extracted, determined, and processed for Western Blot as described previously [8]. The membranes were blocked with 5% skim milk in Tween20/PBS and incubated in the recommended dilution of protein specific antibodies (phospho-ERK-1/2 (#4370), phospho-p38 (#9211), cleaved Caspase-3 (#9664), phospho-NF-κB p65 (#3033), HO-1 (#5141), Bax (#2772) and Bcl-2 (#2870), all Cell Signaling Technology, Danvers, MA, USA) overnight at 4°C. For normalization, blots were re-probed with antibodies to detect total amounts of Caspase-3 (#9665), ERK-1/2 (#4695), p38 (#9212), NF-κB-p65 (#3034, all Cell Signaling Technology, Danvers, MA, USA) and GAPDH (#CSA-335, Enzo Lifesciences, Plymouth, PA, USA). Relative changes in protein expression or phosphorylation in I/R injured retinas either with or without CO were calculated in relation to the corresponding non-ischemic retinas and expressed as “x-fold change versus non-ischemic retina”.

### Electrophoretic mobility shift assay

Electrophoretic mobility shift assays (EMSA) were performed using oligonucleotides containing the consensus binding site for NF-κB (NF-κB consensus sequence 5′-AGT TGA GGG GAC TTT CCC AGG-3′, Promega, Mannheim, Germany) as previously described [8]. Relative DNA-binding activity of NF-κB in I/R injured retinas either with or without CO was calculated in relation to the binding activity in the corresponding non-ischemic retinas and expressed as “x-fold change versus non-ischemic retina”.

### Real time polymerase chain reaction

From retinal tissue harvested at different time points (t = 12, 24, 48 and 72 h), total RNA from ¼ of each retina was extracted using a column-purification based kit (RNeasy Micro Kit, Qiagen, Hilden, Germany) according to the manufacturer's instructions. Reverse transcription was performed with 50 ng of total RNA using random primers (High Capacity cDNA Reverse Transcription Kit, Applied Biosystems, Darmstadt, Germany). Real time polymerase chain reactions (RT-PCR) were done with a TaqMan® probe-based detection kit (TaqMan® PCR universal mastermix, Applied Biosystems, Darmstadt, Germany), using the primers listed in Table 1 (all from Applied Biosystems, Darmstadt, Germany). The PCR assays were then performed on a RT-PCR System (ABI Prism 7000, Applied Biosystems, Darmstadt, Germany) with the following cycling conditions: 95°C for 10 min, 40 cycles of 95°C for 10 sec and 60°C for 1 min. Reaction specificity was confirmed by running appropriate negative controls. Cycle threshold (CT) values for each gene of interest were normalized to the corresponding CT values for GAPDH (ΔCT). Relative gene expression in I/R injured retinal tissue either with CO or room air was calculated in relation to the corresponding gene expression in the non-injured retinal tissue of each individual animal (ΔΔCT).

**Table 1:** Real time polymerase chain reaction primers.

Gene Name	Assay ID
HMOX1	Rn00561387_m1
BAX	Rn02532082_g1
BCL2	Rn99999125_m1
CASP3	Rn00563902_u1
GAPDH	4352338E

Genes of interest and the corresponding assay IDs of real time polymerase chain reactions primers used (HMOX1 = HO-1, heme oxygenase 1; BAX = Bax, Bcl-2-associated X protein; BCL2 = Bcl-2, B-cell lymphoma 2; CASP3 = Caspase-3; GAPDH = glyceraldehyde 3-phosphate dehydrogenase).

### Statistical analysis

Data were analyzed by a computerized statistical program (SigmaPlot Version 11.0, Systat Software Inc., San Jose, CA, USA). The nature of the hypothesis testing was two-tailed. We wished to detect a 50% reduction of RGC death through CO intervention. Based on previously published data and power analysis [8] we assumed that a sample size of n = 8 animals per group would be sufficient to detect such reduction. The results are presented as mean values (±SD) after normal distribution of data had been verified. Two-way ANOVA (RT-PCR, WB and EMSA: Factor A = time with four levels: 12, 24, 48 and 72 h; factor B = intervention with two levels: room air and CO; RGC analysis: Factor A = ischemia with two levels: control and I/R injury; factor B = intervention with four levels: I.: 1. room air, 2. CO immediate, 3. CO 1.5 h and 4. CO 3 h; II.: 1. room air, 2. CO, 3. PD98059 and 4. PD98059+CO) was used for between-group comparisons with post hoc Holm-Sidak test. A p-value<0.05 was considered statistically significant.

## Results

### CO postconditioning suppresses I/R-induced apoptosis

**Figure 1:**
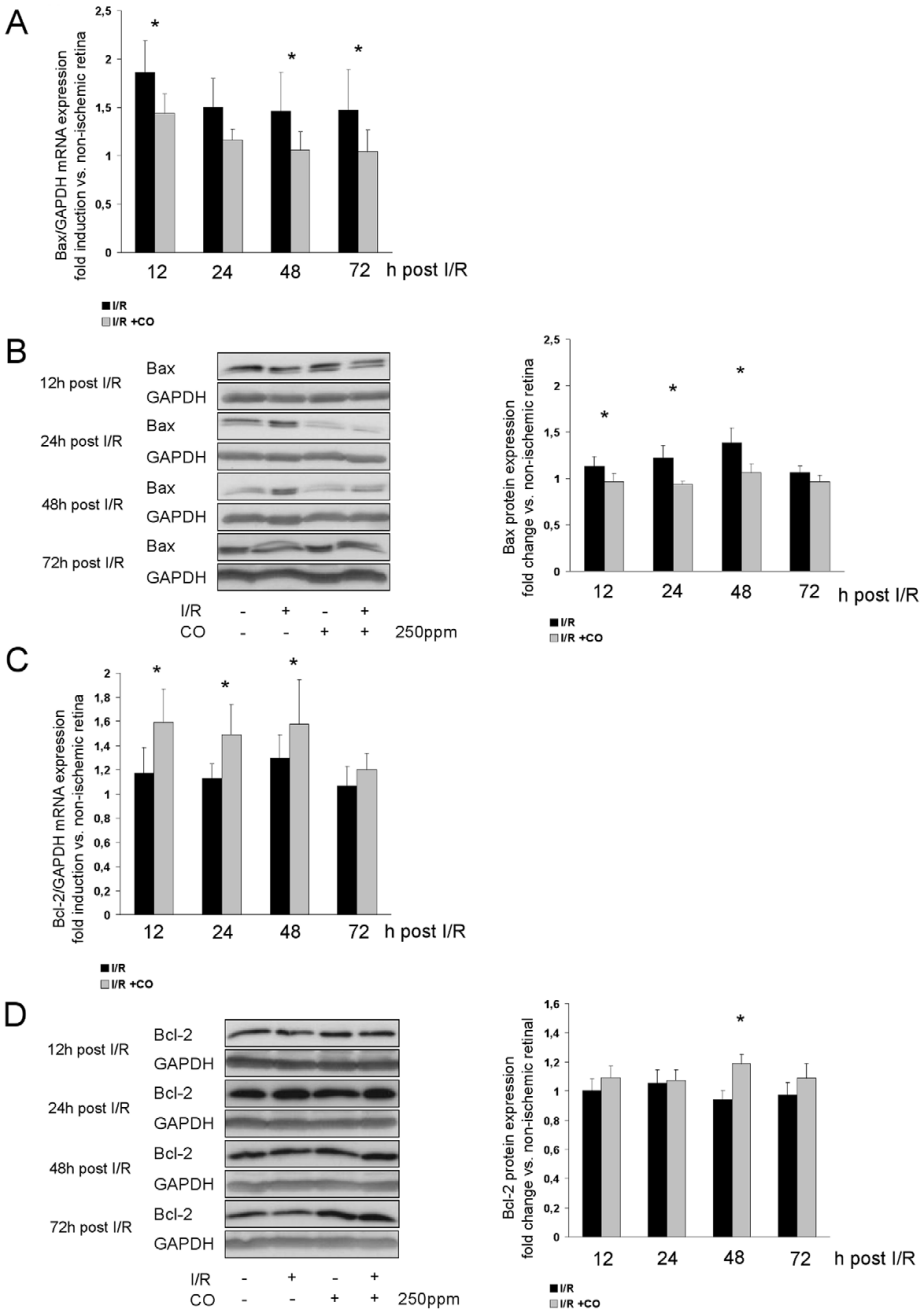
Effects of carbon monoxide postconditioning on retinal Bax and Bcl-2 mRNA and protein expression. (**A**) Fold induction of Bax mRNA expression in ischemic retinal tissue compared to GAPDH in relation to the corresponding non-ischemic retinae analyzed by RT-PCR (n = 8 per group; mean±SD; * p = 0.028, 0.024 and 0.016 I/R vs. I/R+CO at 12, 48 and 72 h). (**B**) Representative Western blot images (of n = 4) analyzing the suppression of retinal Bax protein expression by carbon monoxide postconditioning. Densitometric analysis of n = 4 western blots (mean±SD; * p = 0.028, <0.001 and <0.001 I/R vs. I/R+CO at 12, 24 and 48 h). (**C**) Retinal expression of Bcl-2 mRNA (n = 8 per group; mean±SD; * p = 0.001, 0.011 and 0.038 I/R vs. I/R+CO at 12, 24 and 48 h). (**D**) Representative Western blot images (of n = 4) analyzing the induction of retinal Bcl-2 protein expression by carbon monoxide postconditioning. Densitometric analysis of n = 4 western blots (mean±SD; * p<0.001 I/R vs. I/R+CO at 48 h).

To first answer the question whether CO inhibits I/R-induced retinal apoptosis, mRNA and protein expression of Bax and Bcl-2 were determined. CO postconditioning reduced the retinal mRNA expression of pro-apoptotic Bax at 12, 48 and 72 h (Fig. 1A, I/R 1.9±0.3 vs. I/R+CO 1.4±0.2 fold induction at 12 h; 1.5±0.4 vs. 1.1±0.2 at 48 h and 1.5±0.4 vs. 1.0±0.2 at 72 h) and reduced Bax protein expression at 12, 24 and 48 h (Fig. 1B, I/R 1.1±0.1 vs. I/R+CO 1.0±0.05 fold change at 12 h; 1.2±0.1 vs. 0.9 ±0.03 at 24 h; 1.4±0.2 vs. 1.0±0.1 at 48 h). The expression of anti-apoptotic Bcl-2 mRNA was induced at 12, 24 and 48 h (Fig. 1C, I/R 1.2±0.2 vs. I/R+CO 1.6±0.2 fold induction at 12 h; 1.1±0.1 vs. 1.5±0.1 at 24 h and 1.3±0.2 vs. 1.6±0.3 at 48 h), while protein expression of Bcl-2 was induced at 48 h (Fig. 1D, I/R 0.9±0.1 vs. I/R+CO 1.2±0.1 fold change).

**Figure 2:**
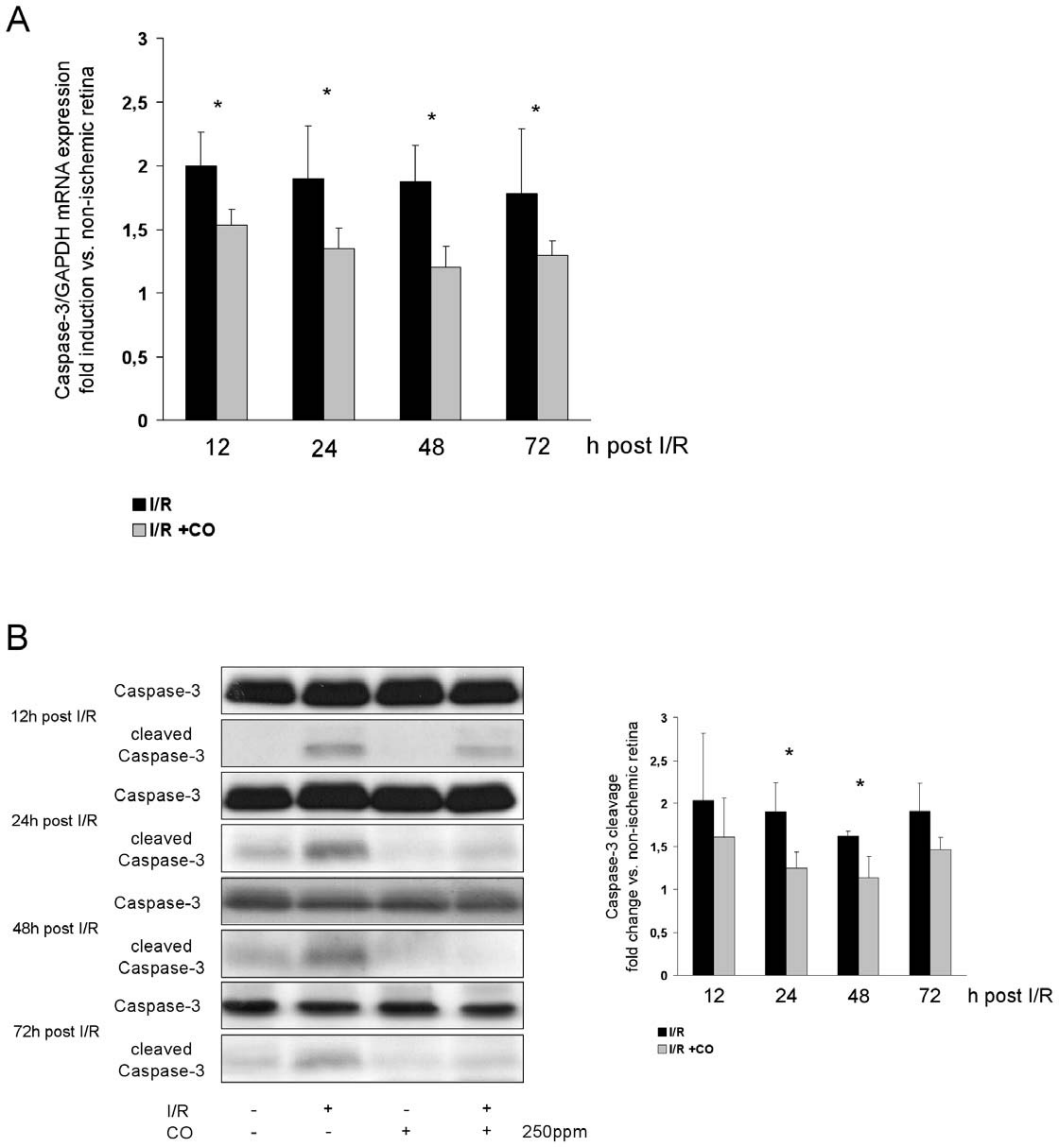
Effect of carbon monoxide postconditioning on retinal expression of caspase-3 mRNA and caspase-3 cleavage. (**A**) Fold induction of caspase-3 mRNA expression in ischemic retinal tissue compared to GAPDH in relation to the corresponding non-ischemic retinae analyzed by RT-PCR (n = 8 per group; mean±SD; * p = 0.007, 0.002, <0.001 and 0.006 I/R vs. I/R+CO at 12, 24, 48 and 72 h). (**B**) Representative Western blot images (of n = 4) analyzing the suppression of retinal cleavage of caspase-3 protein by carbon monoxide postconditioning. Densitometric analysis of n = 4 western blots (mean±SD; p = 0.042 and 0.028 I/R vs. I/R+CO at 24 and 48 h).

To confirm the findings of CO-mediated anti-apoptosis after I/R, we evaluated Caspase-3 expression by RT-PCR and Caspase-3 cleavage using Western blot. Retinal Caspase-3 mRNA expression was reduced when animals inhaled CO after I/R compared to room air inhalation (Fig. 2A, I/R 2.0±0.2 vs. I/R+CO 1.5±0.1 fold induction at 12 h; 1.9±0.4 vs. 1.3±0.1 at 24 h; 1.9±0.3 vs. 1.2±0.2 at 48 h and 1.8±0.5 vs. 1.3±0.1 at 72 h). Cleavage of inactive Caspase-3 was reduced at 24 and 48 h in CO-treated animals compared to room air treated animals (Fig. 2B, I/R 1.9±0.3 vs. I/R+CO 1.2±0.2 fold increase at 24 h; 1.6±0.1 vs. 1.1±0.2 at 48 h).

### CO postconditioning differentially regulates MAP kinase activation

**Figure 3:**
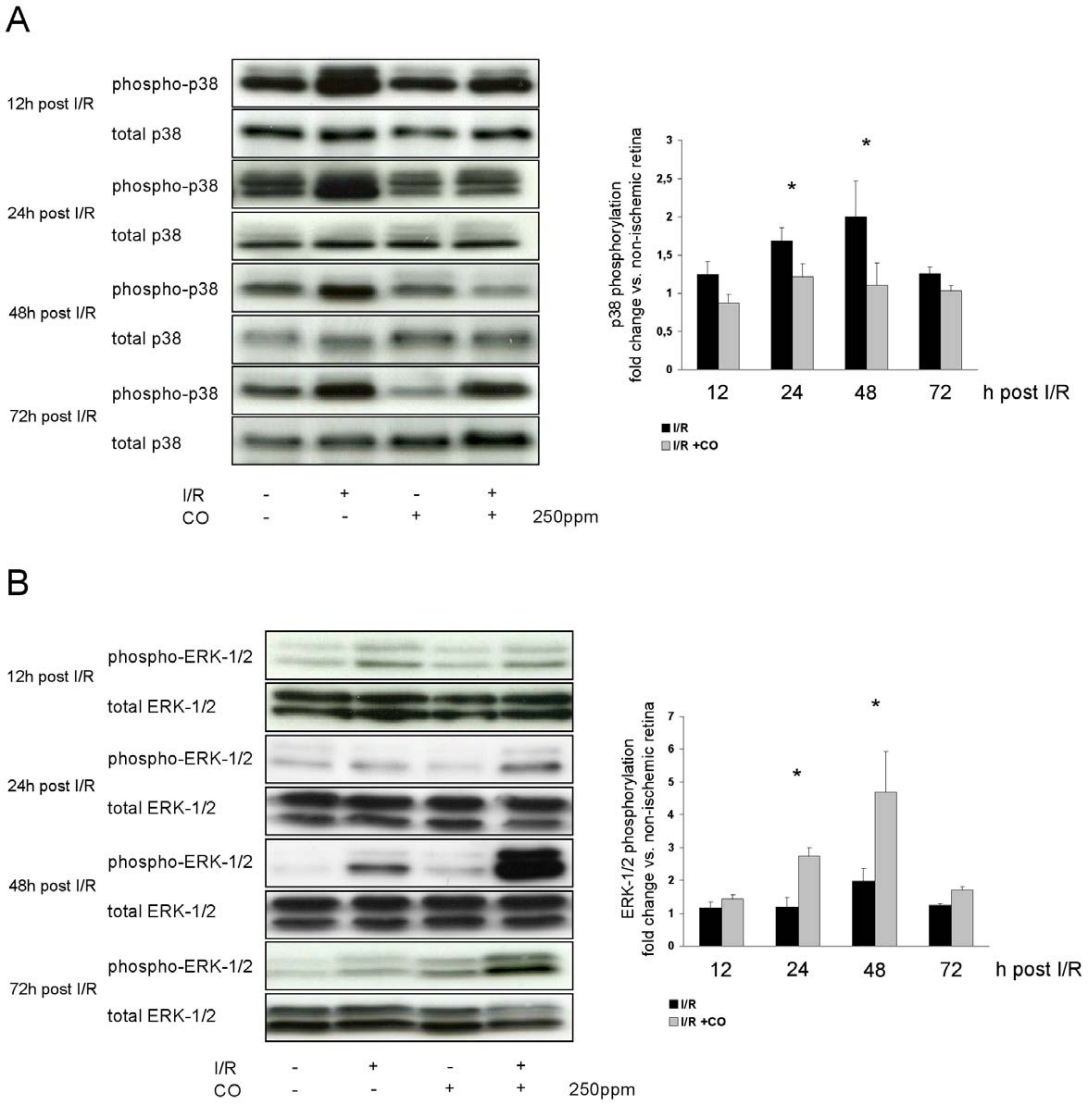
Effects of carbon monoxide postconditioning, inhibiting p38 MAPK while activating ERK-1/2 MAPK phosphorylation. (**A**) Representative Western blot images (of n = 4) analyzing the influence of carbon monoxide postconditioning on the phosphorylation of retinal p38 MAPK. Densitometric analysis of n = 4 western blots (mean±SD; * p = 0.023 and <0.001 I/R vs. I/R+CO at 24 and 48 h). (**B**) Representative Western blot images (of n = 4) analyzing the influence of carbon monoxide postconditioning on the phosphorylation of retinal ERK-1/2 MAPK. Densitometric analysis of n = 4 western blots (mean±SD; * p<0.001 I/R vs. I/R+CO at 24 and 48 h).

Since the MAP kinase pathways play an important role in apoptosis and survival signaling, we next analyzed the effect of CO postconditioning on MAPK activation. While no CO-mediated effect on JNK phosphorylation was detectable during the experiments (data not shown), CO postconditioning after I/R suppressed I/R-induced p38 MAP kinase phosphorylation at 24 and 48 h after I/R (Fig. 3A, I/R 1.7±0.2 vs. I/R+CO 1.2±0.2 fold change at 24 h; 2.0±0.4 vs. 1.1±0.3 at 48 h). In contrast, CO postconditioning increased ERK-1/2 phosphorylation at 24 and 48 h compared to room air treated animals (Fig. 3B, I/R 1.2±0.3 vs. I/R+CO 2.7±0.2 fold increase at 24 h; 1.9±0.4 vs. 4.7±1.2 at 48 h).

**Figure 4:**
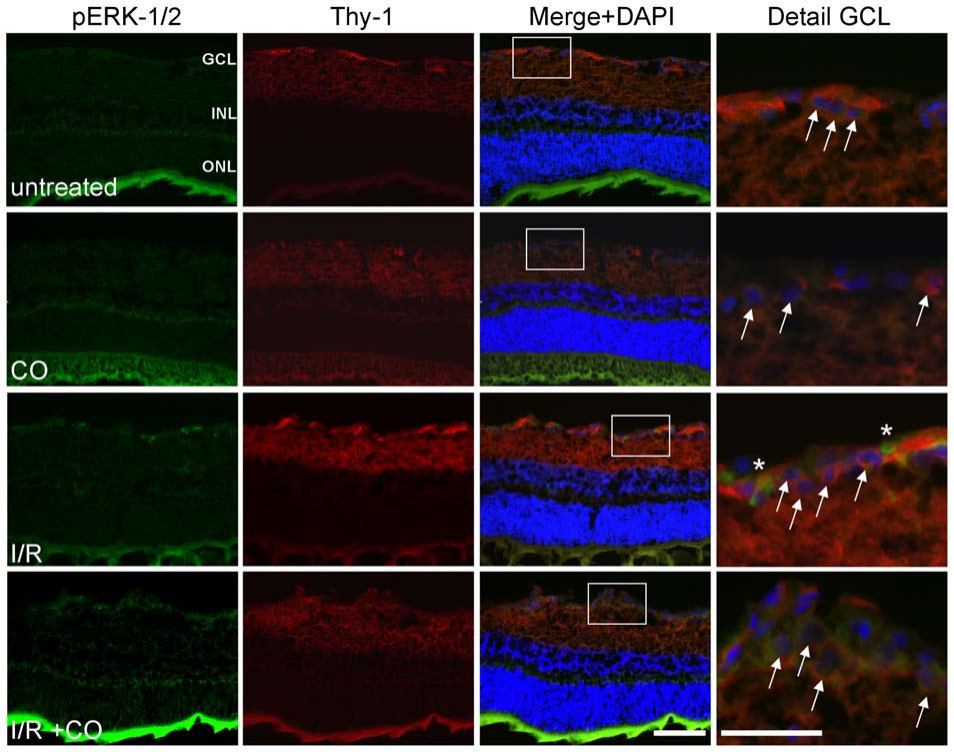
Effect of carbon monoxide postconditioning on phosphorylation of ERK-1/2 in Thy-1 positive RGC. Representative images of immunhistochemical staining against p-ERK-1/2 and Thy-1 in the retina, showing increased phosphorylation of ERK-1/2 in the GCL after I/R and I/R+CO. Only after I/R+CO, ERK-1/2 is phosphorylated in the RGC (white arrows: Thy-1 positive RGC), whereas after I/R alone, p-ERK-1/2 is evident mainly in other cells of the GCL (*: p-ERK-1/2 positive cells). Scale bar 100 µm and 50 µm (in “Detail GCL” pictures). Abbreviations: GCL = ganglion cell layer, INL = inner nuclear layer, ONL = outer nuclear layer, DAPI = 4′,6-diamidino-2-phenylindole.

Dual immunohistochemical staining against Thy-1 (which is exclusively expressed on the surface of RGC) and p-ERK-1/2 demonstrated that ERK-1/2 phosphorylation is detectable in the ganglion cell layer (GCL) after I/R, however, not in RGC (RGC are marked with white arrows in Figure 4, 3^rd^ row, 4^th^ column, p-ERK-1/2-positive cells are marked with *). Inhalation of CO further increased p-ERK-1/2 staining in the GCL, but ERK-1/2 seemed to be phosphorylated predominantly in the RGC itself (white arrows in Fig. 4, 4^th^ row, 4^th^ column).

### CO postconditioning inhibits I/R-induced expression of HO-1

**Figure 5:**
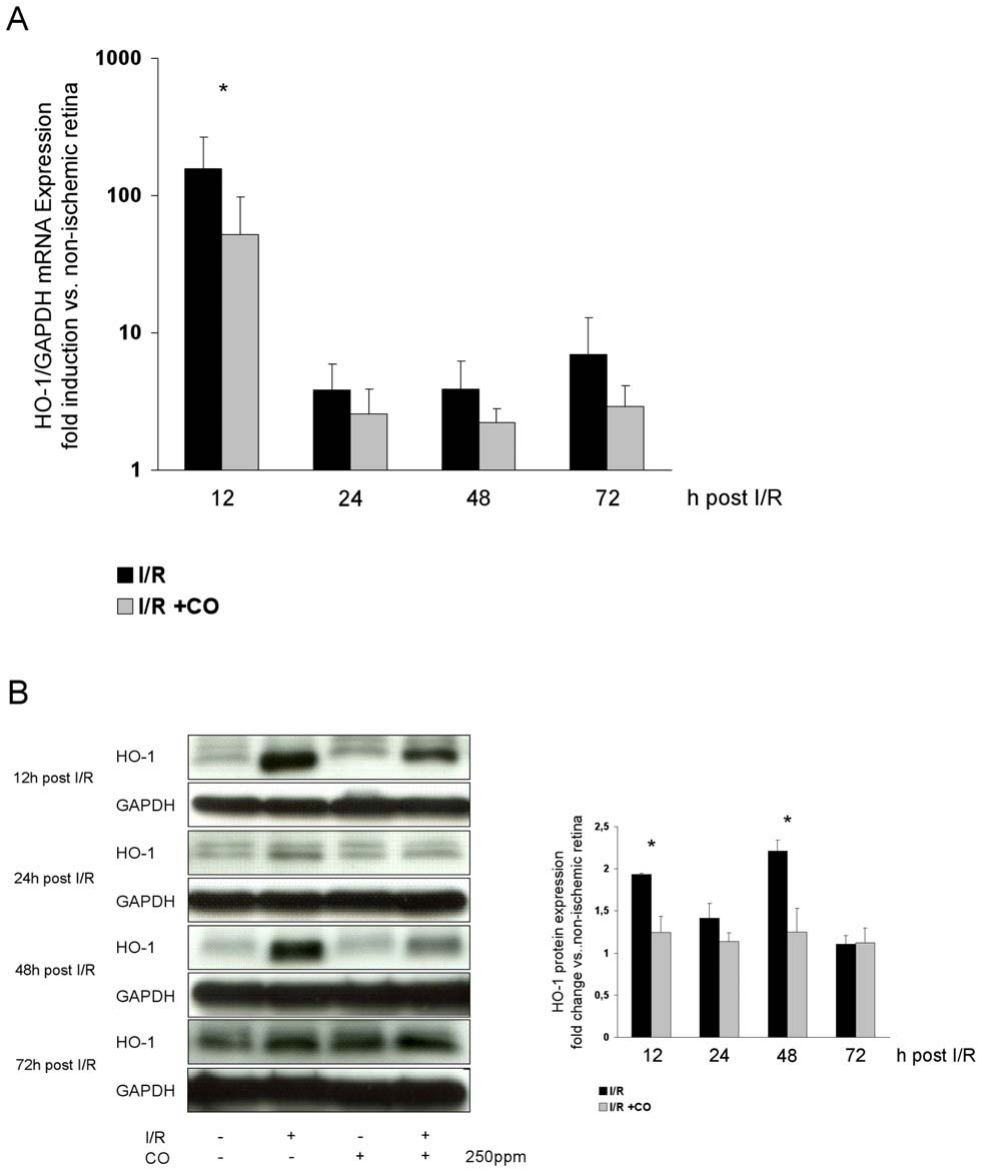
Effect of carbon monoxide postconditioning on retinal expression of HO-1 mRNA and HO-1 protein. (**A**) Fold induction of HO-1 mRNA expression in ischemic retinal tissue compared to GAPDH in relation to the corresponding non-ischemic retinae analyzed by RT-PCR (n = 8 per group; mean±SD; * p<0.001 I/R vs. I/R+CO at 12, logarithmic scale). (**B**) Representative Western blot images (of n = 4) analyzing the suppression of retinal HO-1 protein expression by carbon monoxide postconditioning. Densitometric analysis of n = 4 western blots (mean±SD; * p<0.001 I/R vs. I/R+CO at 12 and 48 h).

To assess the role of HO-1, which is strongly induced during I/R due to inflammatory oxidative cellular stress, we analyzed retinal HO-1 expression after I/R and CO inhalation. Retinal HO-1 mRNA expression (Fig. 5A, I/R 158±107 vs. I/R+CO 52±46 fold induction at 12 h) and HO-1 protein expression (Fig. 5B, I/R 1.9±0.2 vs. I/R+CO 1.2±0.2 fold increase at 12 h; 2.2±0.1 vs. 1.2±0.2 at 48 h) were attenuated by CO inhalation.

### CO postconditioning time-dependently inhibits DNA binding activity of NF-κB

**Figure 6:**
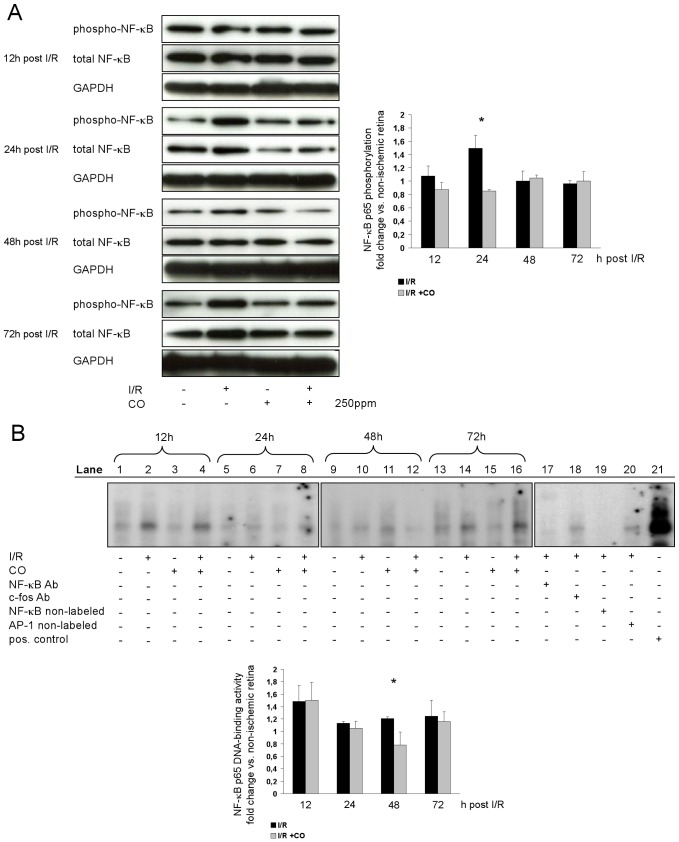
Effects of carbon monoxide postconditioning on NF-κB protein expression, phosphorylation and NF-κB DNA-binding. (**A**) Representative Western blot images (of n = 4) analyzing the influence of carbon monoxide postconditioning on expression and phosphorylation of retinal NF-κB p65. Densitometric analysis of n = 4 western blots (mean±SD; * p<0.001 I/R vs. I/R+CO at 24 h). (**B**) Representative EMSA (of n = 4) of NF-κB DNA binding. Lanes 1–16: individual experiments at 12, 24, 48 and 72 hours after carbon monoxide postconditioning, lanes 17 and 18: supershift analysis shows specificity of NF-κB, lane 19: self competition with unlabeled NF-κB, lane 20: non-self competition with unlabeled AP-1, lane 21: positive control, achieved by induction of SY5Y cell line exposed to PMA/Ionomycin. Densitometric analysis of n = 4 EMSA (mean±SD; * p = 0.016 I/R vs. I/R+CO at 48 h). (Abbreviations: NF-κB Ab = nuclear factor κB antibody, c-fos Ab = c-fos antibody, AP-1 = activator protein 1).

To elucidate the role of NF-κB, a central regulator of inflammation, in CO postconditioning, we performed Western blot analyses. Retinal I/R induced NF-κB phosphorylation at 24 h post I/R, while NF-κB phosphorylation was inhibited in CO-treated animals (Fig. 6A, I/R 1.5±0.2 vs. I/R+CO 0.8±0.1 fold change). Electrophoretic mobility shift assays revealed a CO-mediated inhibition of NF-κB DNA binding at 48 h post I/R (Fig. 6B, I/R 1.2±0.1 vs. I/R+CO 0.7±0.2 fold change).

### CO attenuates glial cell activation in the retina

**Figure 7:**
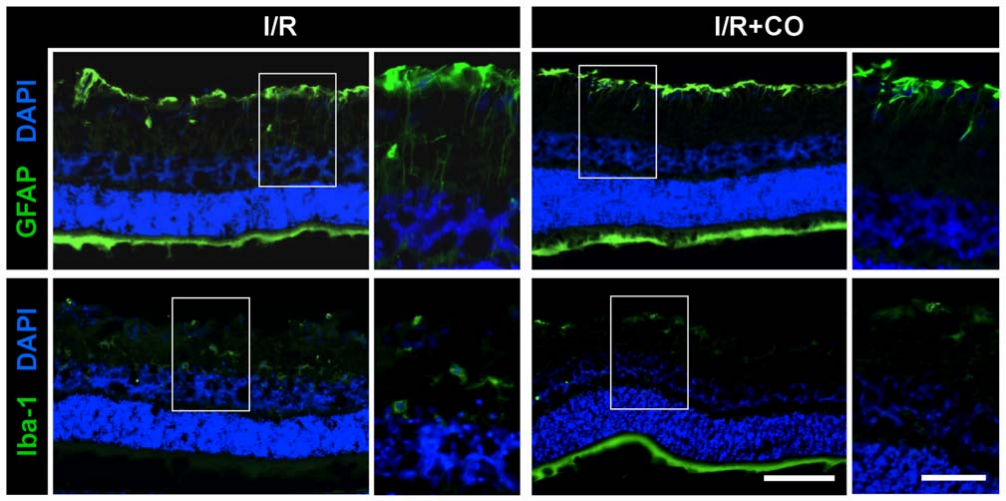
Effect of carbon monoxide postconditioning on glial cell activation in the retina. Representative immunohistochemical GFAP (1^st^ row; Müller cells, “macroglia”) and Iba-1 (2^nd^ row; microglia, macrophages) staining in I/R injured eyes with (right column) or without (left column) CO postconditioning, indicating reduced glial cell activation in the CO-treated retina. Scale bar: 100 µm and 50 µm (in detail picture). Abbreviations: GFAP = Glial fibrillary acidic protein, DAPI = 4′,6-diamidino-2-phenylindole, Iba-1 = ionized calcium binding adaptor molecule 1.

To further confirm these possible anti-inflammatory effects of CO postconditioning, we analyzed glial cell activation in the retina as the hallmark of reactive gliosis and neuroinflammation using GFAP (Müller cells, “macroglia”) and Iba-1 (microglia/macrophages) staining. In the control eyes of room air or CO breathing animals, some baseline GFAP-reactivity and some Iba-1 positive cells were detectable in the GCL and the retina, respectively (data not shown). Compared to control eyes, I/R led to a robust increase in GFAP reactivity indicative of glial cell activation (Fig. 7, left column, upper image). Furthermore, only after I/R Iba-1 positive microglia were detectable throughout all layers of the retina (left column, lower image). CO inhalation after I/R suppressed reactivity for GFAP (right column, upper image) and Iba-1 (right column, lower image).

### CO inhibits ischemia-induced immigration of proliferating cells into the retina

**Figure 8:**
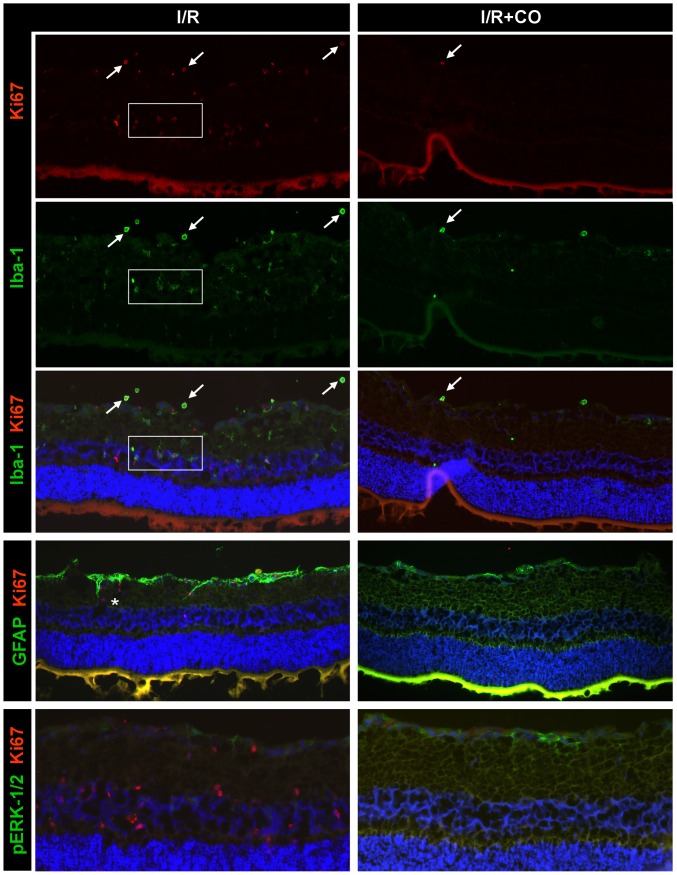
Effect of carbon monoxide postconditioning on immigration of proliferating cells into the retina. Representative immunohistochemical Ki-67 (1^st^ row, dual staining 3^rd^ to 5^th^ row; proliferation marker), Iba-1 (2^nd^ and 3^rd^ row; microglia/macrophage marker), GFAP (4^th^ row; glial cell marker) and p-ERK-1/2 (5^th^ row) staining in I/R injured eyes with (right column) or without (left column) CO postconditioning. White box = Ki-67/Iba-1 positive microglia; White arrows = Ki67/Iba-1 positive macrophages; White * = absent colocalization of GFAP and Ki-67. Abbreviations: GFAP = Glial fibrillary acidic protein, Iba-1 = ionized calcium binding adaptor molecule 1.

We next addressed the question whether I/R and CO influence the proliferation of retinal cells. For this purpose we performed immunhistochemical staining against Ki-67, a marker for proliferating cells. Control eyes showed some weak Ki-67 reactivity only in endothelial cells (data not shown). However, after I/R injury positive signals for Ki-67 were evident throughout the whole retina, but predominantly in the inner retinal layers (Fig. 8, left column, 1^st^ row). This was likely due to immigration of proliferating, Iba-1/Ki-67 positive microglial cells (left column, 2^nd^ and 3^rd^ row; white box). In addition, round Iba-1/Ki-67 positive cells located on top of the GCL were detected (left and right column, 2^nd^ and 3^rd^ row; white arrows). Based on their morphology, these cells were thought to be blood-borne macrophages. Furthermore, cells positive for Ki-67 and negative for Iba-1 (unclassified proliferating cells) and vice versa (non-proliferating microglia/macrophages) were also detectable. CO postconditioning abolished I/R-induced Ki-67/Iba-1 reactivity almost completely (right column, 1^st^ to 3^rd^ row) with nearly no Ki-67 positive microglia and only few macrophages (right column, 1^st^ to 3^rd^ row, white arrow) visible.

In dual GFAP/Ki-67 staining, weak colocalization was detectable (white *, left column, 4^th^ row,), indicating only little proliferation of Müller cells. However, the inhibitory effect of CO postconditioning on I/R-induced Müller cell activation was confirmed (right column, 4^th^ row).

To also investigate a possible link between ERK-1/2 phosphorylation and proliferating cells in the retina, we performed further immunohistochemical studies for p-ERK-1/2 and Ki-67. Ki-67 positive glial cells were not positive for p-ERK-1/2 (left column, 5^th^ row), which was exclusively phosphorylated in the GCL after I/R (left column, 5^th^ row) and further upregulated after I/R +CO (right column, 5^th^ row).

### CO postconditioning reduces I/R-induced death of RGC

**Figure 9:**
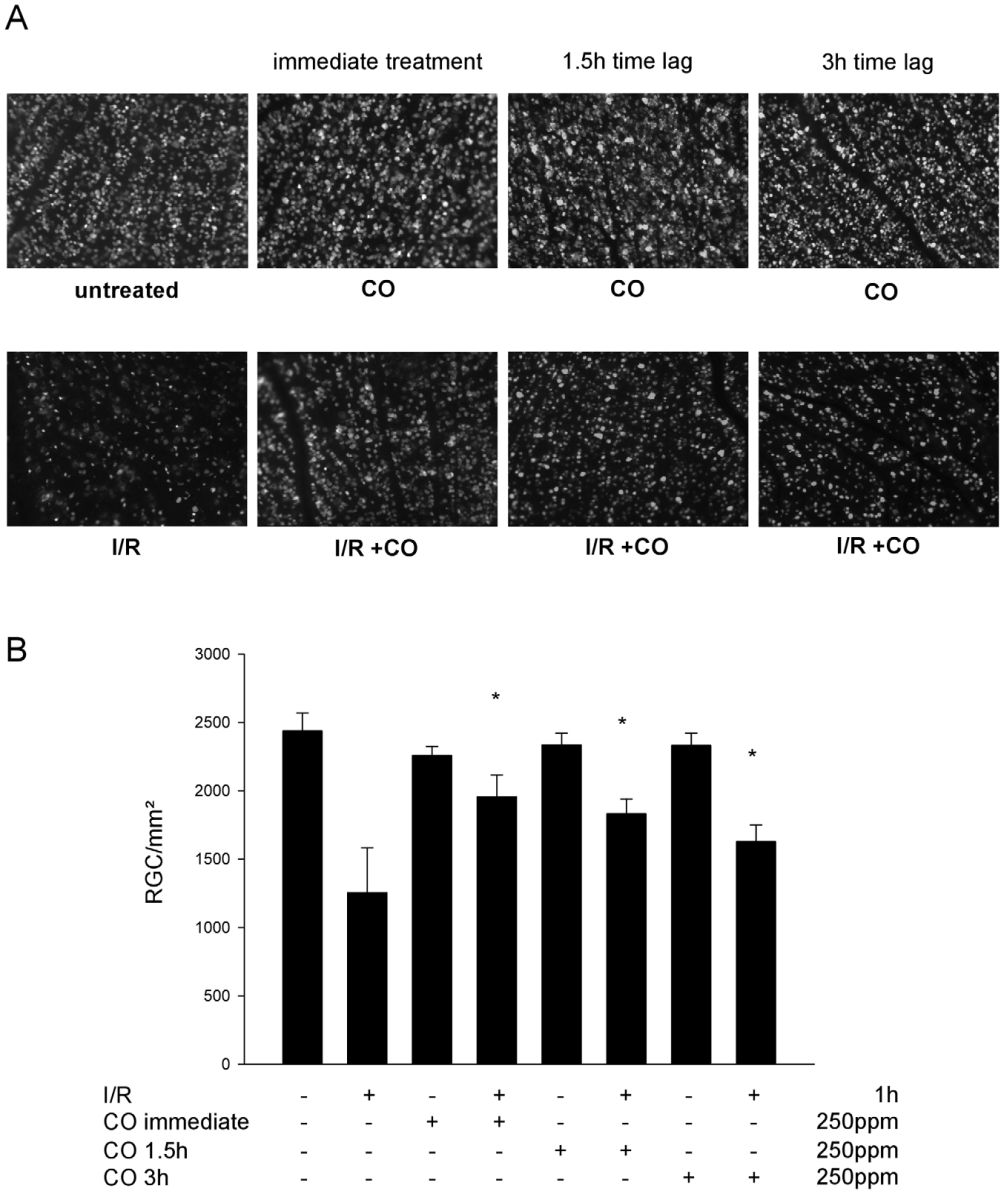
Effect of carbon monoxide postconditioning on ischemia reperfusion (I/R) injury in RGC. (**A**) Representative images (of n = 8) from flat mounts with flourogold-labeled RGC 7 days after I/R injury and CO treatment immediately, 1.5 h and 3 h after initiation of reperfusion. (**B**) Quantification of retinal ganglion cell density [cells/mm^2^] 7 days after I/R injury (n = 8 per group; mean±S.D.; * p<0.001 I/R vs. I/R+CO immediate, vs. I/R+CO 1.5 h and vs. I/R+CO 3 h; IR+CO immediate vs. I/R+CO 3 h).

To answer the question whether these anti-inflammatory and anti-apoptotic effects of CO postconditioning may result in an increase in RGC survival after I/R-injury, we quantified the density of fluorogold-labeled RGC. RGC-densities in the retinas from corresponding control animals did not differ between the different groups (Fig. 9A and 9B: untreated 2437±130 vs. CO immediate treatment 2257±67, CO 1.5 h time lag 2334±88, CO 3 h time lag 2329±94 RGC/mm^2^). I/R injury reduced the RGC-density by approximately 50% (Fig. 9A: lower left image; Fig. 9B: untreated 2437±130 vs. I/R 1255±327 RGC/mm^2^). Postconditioning with CO immediately after I/R injury significantly increased RGC density by ∼50%, resulting in an overall RGC loss of only ∼15% (Fig. 9A: lower left middle; Fig. 9B: I/R 1255±327 vs. I/R+CO 1956±157 RGC/mm^2^). When CO application was delayed for 1.5 and 3 h after initiation of reperfusion, protection was still detectable, yet to a lesser degree, which was significant at 3 h but not at 1.5 h (Fig. 9A: lower right middle and lower right; Fig. 9B: I/R 1255±327 vs. I/R+CO 1.5 h 1830±109 and I/R+CO 3 h 1626±122 RGC/mm^2^).

While above presented results revealed induced phosphorylation of ERK-1/2 MAPK by CO, inhibition of ERK-1/2 activation with the MEK-1/2-inhibitor PD98059 (2 mg/kg) did not attenuate the effect of CO on RGC density (Fig. S1A, lower right image; Fig. S1B: I/R+CO 1956±157 vs. PD98059+I/R+CO 1931±24 RGC/mm^2^).

## Discussion

Treatment of cerebral injury remains difficult. The development of neuroprotective strategies in clinical situations like ischemic or hemorrhagic stroke is under continuous research. Clinical trials failed to provide an improvement of patient outcome. Nevertheless, various experimental data provide evidence that pharmacological and anesthetic agents exhibit neuroprotective properties *in vitro* and *in vivo*.

**Figure 10:**
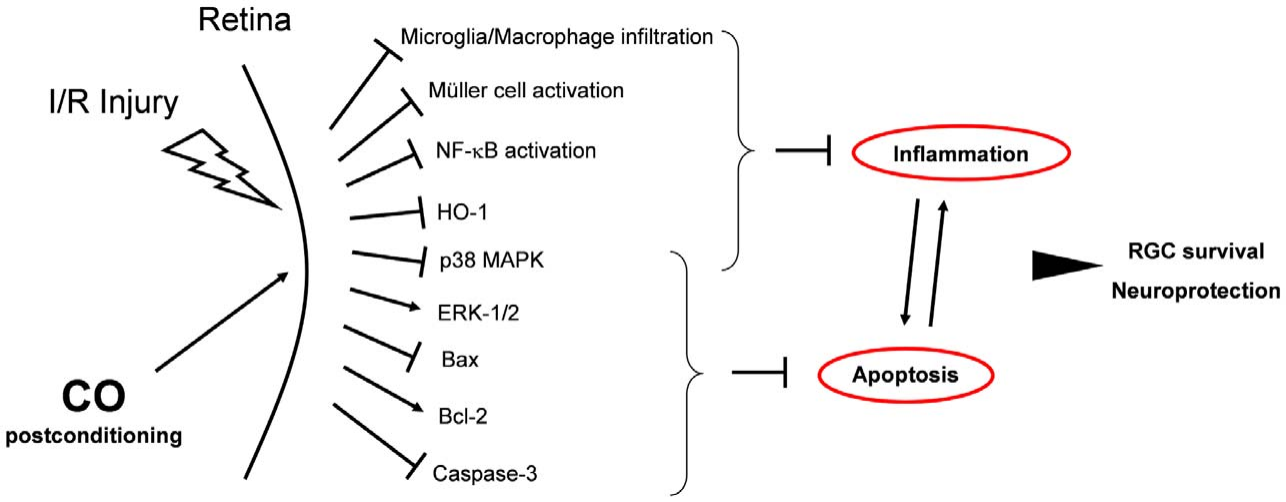
Diagram depicting the proposed mechanism of CO-mediated protective effects on RGC after I/R injury. CO postconditioning after retinal I/R strongly inhibits the inflammatory response by suppressing NF-κB activation, abolishing the infiltration of proliferating microglia and macrophages and inhibiting the activation of Müller cells. Inflammatory oxidative cellular stress is reduced, indicated by inhibition of HO-1 expression. CO attenuates RGC apoptosis as indicated by reduced Caspase-3 activity and Bax expression and by increased Bcl-2 expression. Apoptosis- and inflammation-related MAPK pathways are regulated in favor of anti-apoptosis and anti-inflammation. Overall, these anti-inflammatory and anti-apoptotic effects lead to higher RGC survival and neuroprotection (→ = activation; ⊣ = inhibition).

The main findings of this *in vivo* study in an experimental model of I/R injury can be summarized as follows: (1) Postconditioning with inhaled CO *after* retinal I/R injury inhibits RGC apoptosis indicated by (a) inhibition of Bax/Caspase-3 expression and Caspase-3 cleavage, (b) induction of BCL-2 expression, (c) differential regulation of MAPK pathways which are associated with apoptosis and survival signaling: inhibition of p38 and induction of ERK-1/2. (2) Postconditioning with inhaled CO potently inhibits the inflammatory reaction following I/R since it (a) inhibits NF-κB activation and DNA-binding of NF-κB, (b) inhibits Müller cell activation, (c) abolishes immigration of proliferating microglia and macrophages into the retina and (c) reduces inflammatory oxidative cellular stress. (3) CO postconditioning exerts a significant protective effect on RGC after I/R injury with a “therapeutic window” of at least 3 h for the initiation of CO application after reperfusion. (5) Inhaled CO does not solely act via the ERK-1/2 pathway, since inhibition with a specific inhibitor does not counteract the CO-mediated protective effects. The findings support our hypothesis that CO postconditioning protects neuronal cells *in vivo* via an interdependent network of pathways. A potential interaction of the analyzed pathways is proposed in Figure 10: CO postconditioning after retinal I/R strongly inhibits the inflammatory response and reduces RGC apoptosis, leading to higher RGC survival. However, cause and effect relationships between inflammation and apoptosis have to be investigated in the future.

Previous *in vitro* and *in vivo* studies have demonstrated that CO *preconditioning* exerts neuroprotective effects [7], [8]. However, the concept of *preconditioning* must be questioned with regard to its feasibility and transferability into clinical settings, since patients usually receive medical treatment *after* neuronal ischemic injury [21], [22]. In order to demonstrate CO-mediated organ protection after an injurious event, previous studies demonstrated, that CO *postconditioning* protect the lungs after I/R injury by inhibiting the inflammatory and apoptotic response [23]. However, the effects and the mechanisms of CO postconditioning on neuronal cells *in vivo*, have not been investigated.

In most cases of acute injury deposition of tissue debris is observed due to cell death. In our study, caspase-3 mRNA expression as well as caspase-3 degradation was reduced after CO postconditioning after I/R injury, revealing the anti-apoptotic effects of inhaled CO. In accordance with previous findings, CO postconditioning induced Bcl-2 and suppressed Bax gene expression after I/R injury, demonstrating CO-mediated stabilization of the mitochondrial membrane to prevent cytochrome c release and initiation of apoptosis [24].

The three main members of the MAPK family (p38, ERK-1/2, and JNK) exert different cellular functions depending on the stimulus and timing of activation. Several experimental studies have demonstrated a differential effect of CO on MAPK activation [4], [25]–[27], which in turn resulted in CO-mediated protective effects. Our data also demonstrate a differential effect of CO postconditioning on MAPK activation after I/R injury in retinal cells. Phosphorylation of p38 was suppressed by CO inhalation up to 48 h after I/R injury. Depending on which p38 kinase isoform is predominantly involved, either promotion or inhibition of apoptosis may be fostered [25], [28], [29]. Postconditioning with inhaled CO in our model of I/R seemed to suppress p38 signaling, whereas ERK-1/2 activation was increased in CO-treated animals. Various *in vitro* and *in vivo* studies of retinal I/R injury suggested that activation of the ERK-1/2 pathway mediates protective effects on retinal ganglion cells [8], [30], [31]. However, our data showed that inhibition of ERK-1/2 phosphorylation with the highly selective MEK-1 inhibitor PD98059 did not attenuate the CO-mediated protective effects. This might be due to the fact that various cellular targets are influenced by CO (Fig. 10) and ERK-1/2 is not solely responsible for the observed protective effects. Further experimental *in vivo* studies will be necessary to further elucidate the role of ERK-1/2 in CO-mediated protection of retinal ganglion cells subjected to I/R injury.

Oxidative stress as it occurs during an inflammatory response induces the expression of HO-1 [32]. In our model, I/R injury induced HO-1 expression, whereas CO postconditioning inhibited HO-1 expression at 12 and 48 h after I/R injury. This indicates that CO postconditioning reduces oxidative stress during the inflammatory response following retinal I/R injury.

Inhibition of NF-κB activation represents a molecular mechanism of CO-mediated protection after retinal I/R injury, since activation of NF-κB contributes to neuronal cell death after ischemia, whereas inhibition of NF-κB attenuates retinal ganglion cell death [33]. I/R-induced activation of NF-κB, a central regulator of inflammatory response, was reduced by postconditioning with inhaled CO. In our work, the phosphorylation of p65/p50 heterodimer of NF-κB and the subsequent DNA binding activity was attenuated in animals receiving CO postconditioning 24 and 48 h after I/R injury respectively. These findings are indicative of the anti-inflammatory effects of CO postconditioning.

I/R injury activates glial cells and their reactivity, demonstrated by GFAP staining as an accepted marker of reactive gliosis and neuroinflammation 6 to 72 h after neuronal injury [34]. In addition, GFAP serves as a marker for the activation of retinal Müller cells (“macroglia”) [35], which in turn promote apoptosis in RGC after I/R injury. Inhibition of glial cell activation leads to protection in models of I/R injury in the retina [36]. Our data demonstrate that Müller cell activation and reactive gliosis were inhibited by CO postconditioning, further underlining the CO-mediated anti-inflammatory properties in retinal I/R injury.

Microglial cells are the main effectors of the immune response following CNS injuries, including ischemia. Recent evidence suggests that the activation and immigration of microglial cells may be associated with detrimental and/or beneficial effects on adjacent neurons [37]. In acute injury, microglia has been shown to react within a few hours with a migratory response towards the lesion. Ki-67 antigen is a well-established marker for proliferating cells in the retina [38]. It is a suitable marker for the activation and proliferation of retinal glial cells (Müller cells, microglia) – the hallmarks of reactive gliosis and neuroinflammation – following retinal detachment [39], degenerative disorders [40], laser injury [41] or optic nerve lesion [42]. Ki-67 antigen has also been used as a marker for adult neurogenesis [43] in the brain. By dual staining with Iba-1 we were able to characterize the proliferating cells after I/R as predominantly microglia cells and fewer infiltrating macrophages, since Iba-1 is an established marker for these cells in the retina [44], [45]. The strong increase in Ki-67 and Iba-1 reactivity after I/R is most likely due to immigration of proliferating, non-residential microglia and macrophages. This is probable, since almost no Ki-67 and Iba-1 reactivity was detectable in control eyes and also blood-borne macrophages were visible, indicative of I/R-induced disturbance of the blood-brain barrier with subsequent cellular infiltration. However, further studies will have to address the question whether increases in Ki-67- and Iba-1-reactivity are due to microglia infiltration into the retina or because of activation of residential microglial cells.

Our data demonstrate that CO postconditioning can almost completely abolish the infiltration of proliferating microglia and macrophages, adding strong evidence for the anti-inflammatory effect of CO in retinal I/R injury. Nevertheless, it remains to be investigated in the future, whether activated microglia cells in the retina induce RGC death after I/R themselves or whether these cells are merely attracted to the site of cell death and phagocytosis after the injury. Therefore, it also remains to be investigated whether CO directly inhibits activation of microglia cells or whether this “inhibitory” effect is rather in indicator of CO-mediated neuroprotection via different pathways and less RGC death.

Glial proliferation was not dependent on ERK-1/2 activation, since Ki-67 positive glial cells were p-ERK-1/2 negative. p-ERK-1/2 was predominantly evident in the GCL and the RGC.

The findings of this study are in accordance with our previous ones in the same experimental model of retinal injury, where administration of the same low concentration of inhaled CO before ischemia (i.e., CO preconditioning) was associated with anti-inflammatory, anti-apoptotic and cytoprotective effects [8]. Overall, the degree of cytoprotective response was comparable between preconditioning and postconditioning, with postconditioning representing a realistic treatment option as opposed to preconditioning. Protective effects were detectable up to 7 days after I/R injury. However, no conclusions about the long-term effects on retinal ganglion cells can be drawn from this study.

Delayed onset of CO application 1.5 and 3 hours after I/R injury still exerted protective effects, extending the therapeutic window for CO application into a clinical relevant time period. Attenuation of protection at 3 h is in accordance with previous studies on CO application following neuronal injury [13], indicating that the therapeutic window might end at this time point. However, in another study with the carbon monoxide releasing molecule CORM-3, treatment was only effective either before or 3 days after hemorrhagic neuronal injury [46]. Treatment 3 hours after injury resulted in aggravation of neuronal damage. These differences might be due to different kinetics and *in vivo* distribution of CO gas vs. CORM-3 and due to different models of neuronal injury (I/R vs. hemorrhagic). However, it also demonstrates that in neuronal injury timing of CO treatment is crucial.

The use of a potentially toxic gas must be carefully weighed. In this study, we used lower concentrations compared to human studies, which examined the effects of continuous carbon monoxide inhalation on carboxyhemoglobin levels. For example, volunteers breathed CO concentrations of up to 1000 ppm until their carboxyhemoglobin levels reached 10 to 12% and were then assigned to hyperbaric oxygen therapy [47]. A clinical study by Mayr et al. showed no clinical signs of CO toxicity after exposure of 250 and 500 ppm [48]. Modest increases in carboxyhemoglobin levels equivalent to that resulting from cigarette smoking do not have any appreciable acute sympathetic and hemodynamic effects in healthy humans. Furthermore, the concentrations used here are comparable to the levels used in humans (0.03%) during measurement of DLCO (lung diffusion capacity for carbon monoxide), a standard pulmonary function test [49].

In conclusion, the present study in a model of neuronal injury demonstrates that postconditioning with inhaled CO protects retinal ganglion cells against I/R injury and cellular destruction. Possible mechanisms for these neuroprotective properties are the inhibition of microglia and macrophage infiltration leading to reduced neuroinflammation or the direct inhibition of RGC apoptosis. Future pharmacological or genetic interventions are necessary to further elucidate the distinct role of these pathways in terms of their relevance and interdependence in CO-mediated protective effects. In the future, CO might be a treatment option for acute ischemic injury to the retina and the brain.

## Supporting Information

Figure S1 **Effect of ERK-1/2 inhibition on CO-mediated protection.** (**A**) Representative images (of n = 8) from flat mounts with flourogold-labeled RGC 7 days after I/R injury, CO postconditioning treatment and/or ERK-1/2 inhibition with PD98059. (**B**) Quantification of retinal ganglion cell density [cells/mm^2^] 7 days after I/R injury, CO postconditioning treatment and/or ERK-1/2 inhibition with PD98059 *in vivo* (n = 8 per group; mean±S.D.; * p<0.001 I/R vs. I/R+CO and I/R+CO vs. I/R+PD98059).
(TIF)
