# Supplementary figures and images for: Postconditioning with Inhaled Carbon Monoxide Counteracts Apoptosis and Neuroinflammation in the Ischemic Rat Retina
Source: PLoS One. 2012 Sep 28;7(9):e46479. doi: 10.1371/journal.pone.0046479 (PMC3460901; doi:10.1371/journal.pone.0046479)

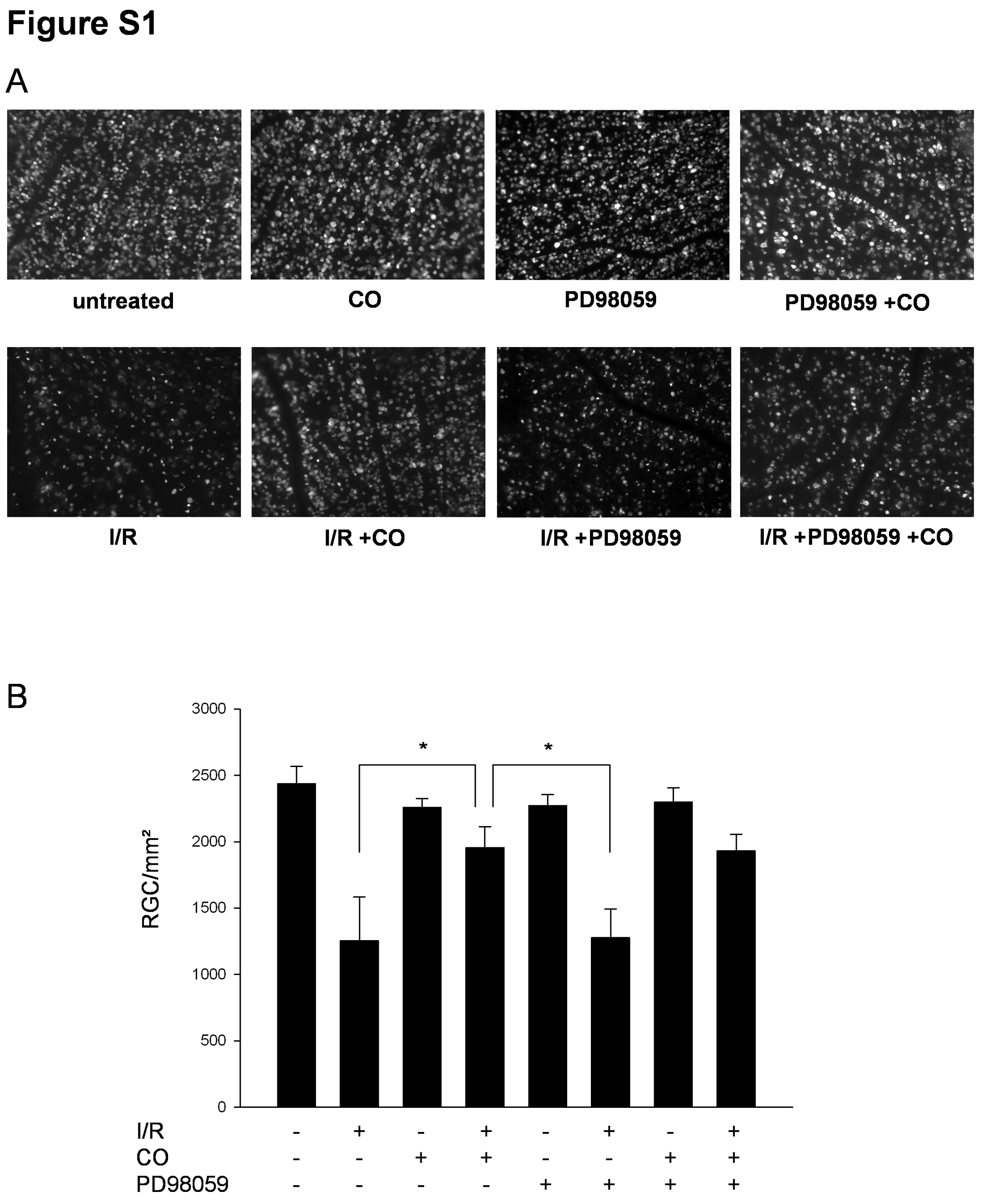

Supplement: Figure S1 — Effect of ERK-1/2 inhibition on CO-mediated protection. (A) Representative images (of n = 8) from flat mounts with flourogold-labeled RGC 7 days after I/R injury, CO postconditioning treatment and/or ERK-1/2 inhibition with PD98059. (B) Quantification of retinal ganglion cell density [cells/mm2] 7 days after I/R injury, CO postconditioning treatment and/or ERK-1/2 inhibition with PD98059 in vivo (n = 8 per group; mean±S.D.; * p<0.001 I/R vs. I/R+CO and I/R+CO vs. I/R+PD98059). (TIF) [file pone.0046479.s001.tif]
